# Novel Hydrolytic Degradable Crosslinked Interpenetrating Polymeric Networks (IPNs): An Efficient Hybrid System to Manage the Controlled Release and Degradation of Misoprostol

**DOI:** 10.3390/gels9090697

**Published:** 2023-08-29

**Authors:** Yasir Mehmood, Hira Shahid, Kashif Barkat, Numera Arshad, Akhtar Rasul, Mohammad N. Uddin, Mohsin Kazi

**Affiliations:** 1 Department of Pharmaceutics, Faculty of Pharmaceutical Sciences, Government College University Faisalabad, Faisalabad P.O. Box 38000, Pakistan; akhtar.rasul@gcuf.edu.pk; 2 Riphah Institute of Pharmaceutical Sciences (RIPS), Riphah International University Faisalabad, Faisalabad P.O. Box 38000, Pakistan; 3 Department of Pharmacology, Faculty of Pharmaceutical Sciences, Government College University Faisalabad, Faisalabad P.O. Box 38000, Pakistan; hirashahid16@yahoo.com; 4 Faculty of Pharmacy, The University of Lahore, Lahore P.O. Box 54000, Pakistan; kashif.barkat@pharm.uol.edu.pk; 5 Department of Pharmacy, COMSATS University Islamabad, Lahore Campus, Lahore P.O. Box 54000, Pakistan; drnumeraarshad@cuilahore.edu.pk; 6 College of Pharmacy, Mercer University, 3001 Mercer University Drive, Atlanta, GA 30341, USA; uddin_mn@mercer.edu; 7 Department of Pharmaceutics, College of Pharmacy, King Saud University, P.O. Box 2457, Riyadh 11451, Saudi Arabia

**Keywords:** misoprostol, interpenetrating networks, crosslinker, pH responsiveness, hydrogels

## Abstract

Purpose: The goal of this study was to make pH-sensitive HPMC/Neocel C19-based interpenetrating polymeric networks (IPNs) that could be used to treat different diseases. An assembled novel carrier system was demonstrated in this study to achieve multiple functions such as drug protection and self-regulated release. Methods: Misoprostol (MPT) was incorporated as a model drug in hydroxyl-propyl-methylcellulose (HPMC)- and Neocel C19-based IPNs for controlled release. HPMC- and Neocel C19-based IPNs were fabricated through an aqueous polymerization method by utilizing the polymers HPMC and Neocel C19, the initiator ammonium peroxodisulfate (APS), the crosslinker methylenebisacrylamide (MBA), and the monomer methacrylic acid (MAA). An IPN based on these materials was created using an aqueous polymerization technique. Samples of IPN were analyzed using scanning electron microscopy (SEM), atomic force microscopy (AFM), differential scanning calorimetry (DSC), thermal analysis (TGA), and powder X-ray diffraction (PXRD). The effects of the pH levels 1.2 and 7.4 on these polymeric networks were also studied in vitro and through swelling experiments. We also performed in vivo studies on rabbits using commercial tablets and hydrogels. Results: The thermal stability measured using TGA and DSC for the revised formulation was higher than that of the individual components. Crystallinity was low and amorphousness was high in the polymeric networks, as revealed using powder X-ray diffraction (PXRD). The results from the SEM analysis demonstrated that the surface of the polymeric networks is uneven and porous. Better swelling and in vitro results were achieved at a high pH (7.4), which endorses the pH-responsive characteristics of IPN. Drug release was also increased in 7.4 pH (80% in hours). The pharmacokinetic properties of the drugs showed improvement in our work with hydrogel. The tablet MRT was 13.17 h, which was decreased in the hydrogels, and its AUC was increased from 314.41 ng h/mL to 400.50 ng h/mL in hydrogels. The blood compatibility of the IPN hydrogel was measured using different weights (100 mg, 200 mg, 400 mg, and 600 mg; 5.34%, 12.51%, 20.23%, and 29.37%, respectively). Conclusions: As a result, IPN composed of HPMC and Neocel C19 was successfully synthesized, and it is now possible to use it for the controlled release of MPT.

## 1. Introduction

The use of cutting-edge technologies to deliver various therapeutic agents has compelled researchers to create a variety of innovative drug delivery systems. The active ingredient ratio has been substantially maintained within the therapeutic range by delivering active agents to specified areas at controlled rates in accordance with physiological needs [1]. Potential drug delivery technologies include liposomes, nanoparticles, dendrimers, niosomes, microspheres, microneedles, micelles, and hydrogels [2].

Due to the way their hydrophilic polymeric systems work, hydrogel interpenetrating networks (IPNs) have been the most successful method of drug delivery so far. Hydrogels are made up of a polymeric network with a number of functional groups, like amino, carboxylic, and hydroxyl groups. These groups give these substances certain properties, like the ability to absorb water. The delivery strategy of active medicines can have a large impact on their therapeutic potential. Polymeric network carriers have recently received a large amount of attention because changes could be made to their polymeric network systems that could lead to a controlled and targeted release of the active moiety they carry. Additionally, the polymeric networks of such systems keep the active moiety from straying beyond the therapeutic window [3]. Among the numerous kinds of polymeric carriers, responsive hydrogels have been demonstrated to be one of the most essential drug delivery strategies. These have proven to be effective in the delivery of a wide range of therapeutic substances for diagnostic as well as therapeutic purposes, including genes for the purposes of diagnosis and tissue engineering. They are sensitive to chemical, physical, and biological stimuli [4].

Due to variations in body pH during normal and disease states, pH responsiveness has led to more in-depth self-monitoring of therapeutic agent delivery devices [5]. Interferon, insulin, doxorubicin, and dexamethasone are some examples of the pH-sensitive polymeric systems that have been created relatively recently for the treatment of ophthalmologic disorders, as well as cancer and diabetes.

Misoprostol, a prostaglandin analog and uterotonic, is used to treat and prevent postpartum hemorrhage (PPH) and is listed among the essential drugs by the World Health Organization (WHO) [6]. In low-income nations, postpartum hemorrhage (PPH) is the main reason for maternal death. An amount of 500 mL or more of blood is lost within 24 h of delivery, according to the definition [7]. Oxytocin is the first-choice medication for PPH prophylaxis and therapy; however, it is quite sensitive to environmental influences. It must normally be kept between 2 °C and 8 °C to avoid degradation [8,9,10,11]. This storage demand may be challenging, particularly in rural parts of low- and middle-income countries (LMICs), where there is a lack of infrastructure and ambient temperatures are usually high [12,13,14]. In fact, a number of earlier investigations have demonstrated that the quality of oxytocin, particularly in LMICs, is frequently subpar [14,15]. Misoprostol comes in pill form, making it convenient for oral administration without the requirement for refrigeration. Therefore, misoprostol tablets seem to provide a convincing alternative to oxytocin injections for the prevention and treatment of PPH in settings where trained personnel are not always available to administer medications parenterally or where suitable storage conditions for oxytocin cannot be guaranteed [16]. Misoprostol is also used to induce labor, induce abortions, treat and prevent ulcers brought on by the use of nonsteroidal anti-inflammatory medicines (NSAIDs), and treat and prevent ulcers brought on by NSAID use [17].

There are some significant degradation issues in all marketed tablets which can degrade after some months [17]. Due to these degradations, some kinds of impurities are generated within tablets, and these can be hazards to patients. The shelf life of these tablets is generally two years, and throughout the shelf life, tablets cannot maintain their potency. To overcome this issue, we tried to prepare misoprostol hydrogels and at the same time we controlled the drug release so that patients can take one at a time.

Blends of controlled-release hydrogels primarily contain isolated natural polymers. Neocel C19 is a codried mixture of sodium carboxymethylcellulose and microcrystalline cellulose. Neocel C19 undergoes two alterations that are derived from starch: the first is substitution, which increases hydrophilicity; the second is crosslinking, which decreases water solubility and causes gel formation [18]. When it comes into contact with a solution, the polar polymer known as HPMC, which is only slightly soluble in water, undergoes swelling, which ultimately leads to the formation of a gel mass [19]. In addition to this, methacrylic acid, also known as MAA, is a polymer that is hydrophilic. In reaction to changes in pH, MAA undergoes ionization and deionization, which is further explained by the presence of carboxylic acid (-COOH) in its chemical structure. This uniqueness further leads to swelling within water [20]. In the current research, various ratios of Neocel C19 were chemically crosslinked with HPMC through a crosslinker (methylene bisacrylamide) to formulate pH-responsive HPMC- and Neocel C19-based IPN hydrogels. These IPN hydrogels were synthesized to deliver the loaded model drug (MPT) in a controlled-release manner.

## 2. Results and Discussion

Neocel C19 is a biodegradable, natural, and biocompatible polymer. After oral use, Neocel C19 can dissolve quickly due to its poor mechanical strength. This limitation was solved by crosslinking Neocel C19 with different concentrations with a constant concentration of HPMC in the presence of MAA, which strengthened the structure of the Neocel C19 and increased its strength. We discovered that the solution became incredibly viscous when the Neocel C19 concentration increased and drug entrapment also increased. Additionally, we discovered that using a low Neocel C19 concentration led to the creation of hydrogels that were distorted. A two-percent concentration of Neocel C19 was found to be ideal.

### 2.1. Determination of Drug Entrapment Efficiency

Using a mortar and pestle, MPT-loaded hydrogels were smashed/crushed and weighed. All the drugs were extracted from the crushed hydrogels by using the buffer. The solution was centrifuged, and the supernatant was collected. By setting the maximum wavelength at 200 nm on an HPLC, the clear supernatant solution was analyzed with the mobile phase to determine the quantity of MPT [21]. Through the following equation, the entrapment efficiency of formulated hydrogels for MPT was estimated [22]. All the formulations, NH-1 to NH-5, were checked using the same process. The results are mentioned in the Table 1 about drug entrapment. Maximum drug entrapment was found in the NH-5 formulation in which we had used the maximum quantity of Necocel C19. We selected this formulation for further characterizations.

### 2.2. Method Development and Optimization for Pharmacokinetic Study

**Figure 1:**
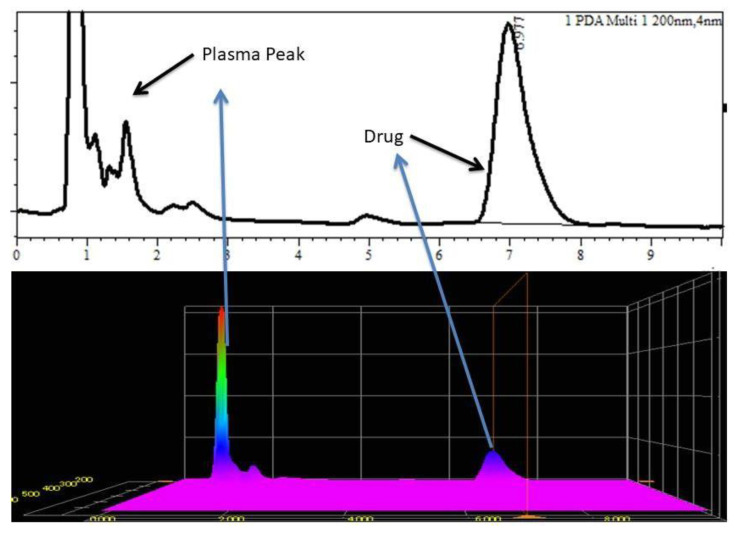
Chromatogram of drug with plasma spiking (3D graph).

A stock solution was prepared and spiked with plasma for method validation parameters such as accuracy, linearity, precision, and recovery. To prepare working standard solutions, the drug stock solution was serially diluted with the mobile phase. Plasma spiking was performed by adding 20 µL of the working standard solution to 100 µL of blank plasma from rabbits. Final concentrations of 20, 50, 100, 200, and 500 ng/mL were prepared with the mobile phase after extraction with an organic solvent. The R^2^ value was larger than 0.996, and the linear regression equation was y = 0.0019 × 0.0032 (Table 2). Excellent linearity over the concentration range (20–500 ng/mL) was proven. The limits of quantification and detection (LOQ and LOD) were 10 and 5 ng/mL, respectively. The recovery values for the drug (n = 3) were determined to be 92.2% ± 3.1%, 89.2% ± 2.0%, and 89.5% ± 3.2%. The drug had an intraday accuracy range of 94.1% to 102.3% and an interday accuracy range of 96.2% to 97.4% (Table 1). In rabbit plasma samples, the intraday and interday precisions for the drug were found to be less than 7.2% and 9.4%, respectively, confirming the excellent precision of the established approach. Figure 1 shows the plasma peak and drug peak in the chromatograph at 500 ng/mL, and during the performance, no interference was seen.

### 2.3. Scanning Electron Microscopy and Investigation of Surface Roughness with AFM

**Figure 2:**
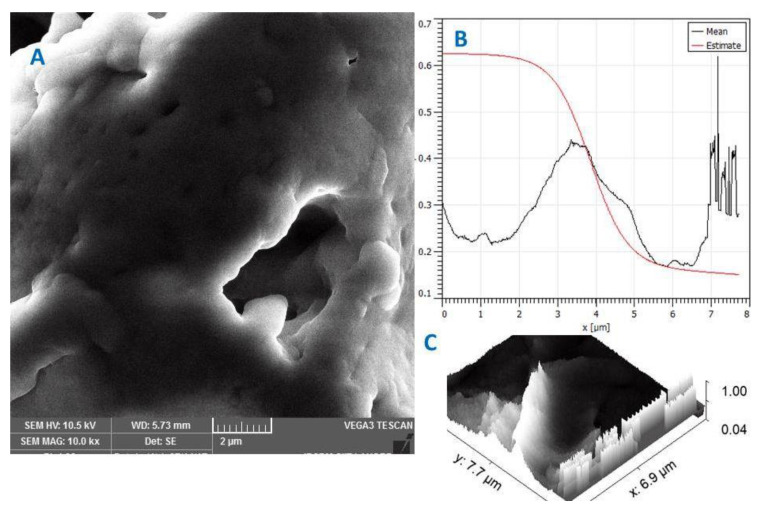
(**A**) SEM and (**B**,**C**) AFM images of the IPN hydrogel.

By using scanning electron microscopy (SEM) analysis, the morphological characteristics of the IPN hydrogel were identified. An IPN hydrogel micrograph at one magnification is shown in Figure 2A. In the SEM analysis, the IPN hydrogel composed of HPMC and Neocel C19 can be seen to have a porous and uneven surface (Figure 2A). In addition, cracks are visible all over the uneven surface. It is possible that these cracks are the result of a drying treatment and the shrinkage of the polymeric system. These pores and fissures allow the solvent to enter the IPN hydrogels, causing swelling and the subsequent release of MPT. The inclusion of Neocel C19 improved the porosity and surface roughness of the hydrogel samples, according to the results of structural and microscopic examination of the samples. One of the most important categories of hydrogels is made up of materials with pores that aid in enclosing medications. The surface roughness of the NH-5 hydrogel is shown with AFM in Figure 2B,C. By examining the physicochemical environment of cavities, special characteristics can be provided in the interaction with guest molecules, and the structure can be modified for additional uses.

### 2.4. Fourier Transform Infrared Spectroscopy

**Figure 3:**
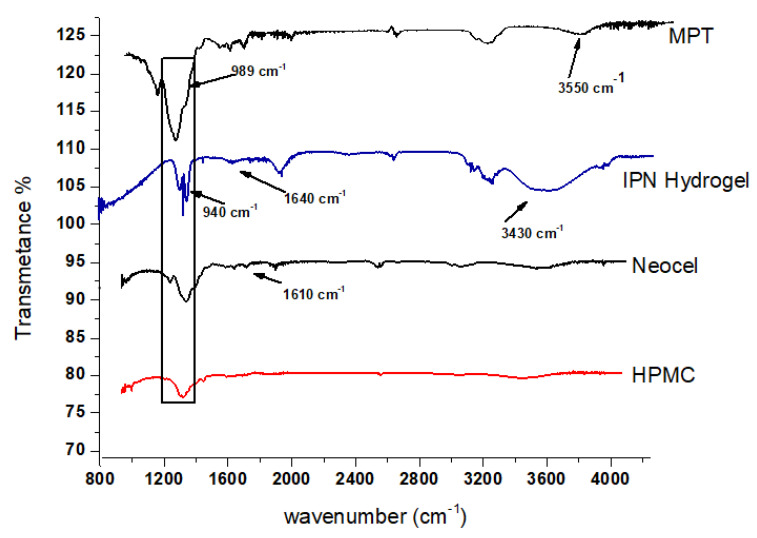
FTIR spectra of MPT, IPN hydrogel, Neocel C19, and HPMC.

Figure 3 displays the FTIR spectra of the MPT drug, loaded IPN hydrogel, and polymers (Neocel C19 and HPMC). The 800–4000 cm^−1^ scanning range was employed to analyze all of the crushed materials.

The majority of the eight chemical bonds in misoprostol were C=O (ester), C=O (carbonyl bond), C-OH (alcohol), C=C (alkene), C-C (alkane), C-H (aliphatic), and CH_3_. The study’s peak measurements were 3550 cm^−1^ O-H (alcohol), 1737 cm^−1^ C=O (ester), 1364 cm^−1^ CH_3_, and 898 cm^−1^ C-C (alkane) [23].

The stretching frequencies of the OH, C-H, and C-O bonds/groups were observed in the FTIR spectra of the HPMC band at 3456 cm^−1^, 2932 cm^−1^, and 1065 cm^−1^, respectively. On the other hand, the bending vibration of the OH groups on the HPMC is demonstrated at 1381 cm^−1^ [24].

With respect to the Neocel C19 spectrum, at 3600 cm^−1^, a broad band revealed the OH stretching group of the molecule. Bands at 1610 cm^−1^ reflect stretching (symmetric and asymmetric) of the C-O-C group.

In the FTIR of the IPN hydrogel, a small peak at 940 cm^−1^ represents the stretching vibration of C-C (alkane). In the range of 1640 cm^−1^, distinct bands of C=O (ester) were observed. One more peak was observed in the IPN hydrogel at 3430 cm^−1^ O-H (alcohol). All described peaks were also observed in the MPT pure compound, which indicated successful drug loading in the IPN hydrogel. Peaks of individual components showed minor shifting, which may be due to crosslinking of polymeric chains. These peaks of crosslinking confirmed the formation of a polymeric network.

### 2.5. Thermal Analysis

**Figure 4:**
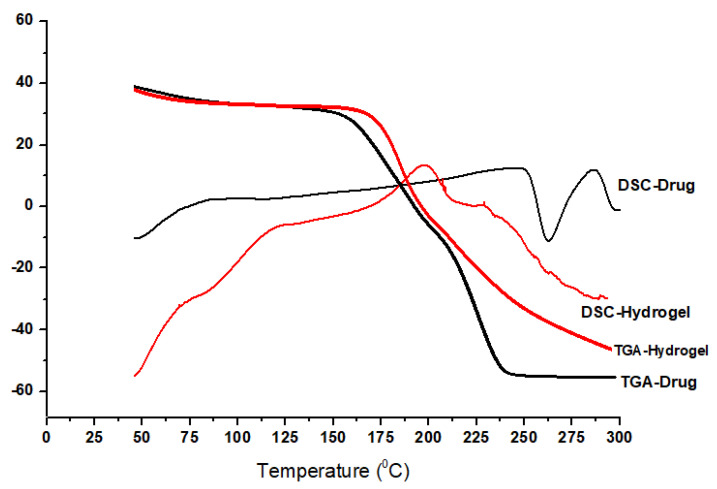
DSC and TGA of the drug and IPN hydrogel.

Figure 4 displays the reaction of IPN formulations with pure MPT and MPT loaded against heat flow. Moisture loss is represented by a slight exothermic curve at approximately 70 °C in a pure MPT DSC graph. The peak at 250 °C shows a breach in the link between the melting point of the polymeric network and the network’s polymer [25]. The DSC of IPN formulations displayed a thermogram with a broad peak from 50 °C to 150 °C, which represents moisture loss and eventually causes bond breaking within the polymeric system. The main exothermic peaks were observed at 200 °C, which was due to polymeric networking degradation. A small curve was also visible in the IPN hydrogel, which was sustained at 300 °C and showed the thermal breakdown of the polymeric network. The thermogram clearly shows that MPT is more thermally stable in a hydrogel formulation than it is as a pure medication.

The MPT TGA curve (Figure 4) demonstrated a weight decrease in various stages. An initial loss of mass occurred between temperatures of 50 °C and 90 °C, and this was mostly due to the evaporation of water molecules. The second loss, which occurred between 150 °C and 300 °C, was brought on by the molecule of MPT’s instability, which resulted in thermal deterioration. A total of 70% of the MPT drug mass was lost. It assumed a significant weight loss of 70% during the second stage (from 150 °C to 300 °C) [26].

TGA of the IPN hydrogel with MPT loading revealed mass losses in two phases. A very similar pyrolytic pattern was demonstrated by the MPT medication that contained the crosslinking ingredient, with just minor variations in the breakdown profile. A 5% weight loss was observed from 50 to 160 °C, and a 40% weight loss was observed at 300 °C. In order to demonstrate its crosslinking, the IPNA hydrogel samples were made to be thermally more stable than the pure drug.

### 2.6. Powder X-ray Diffraction Analysis

**Figure 5:**
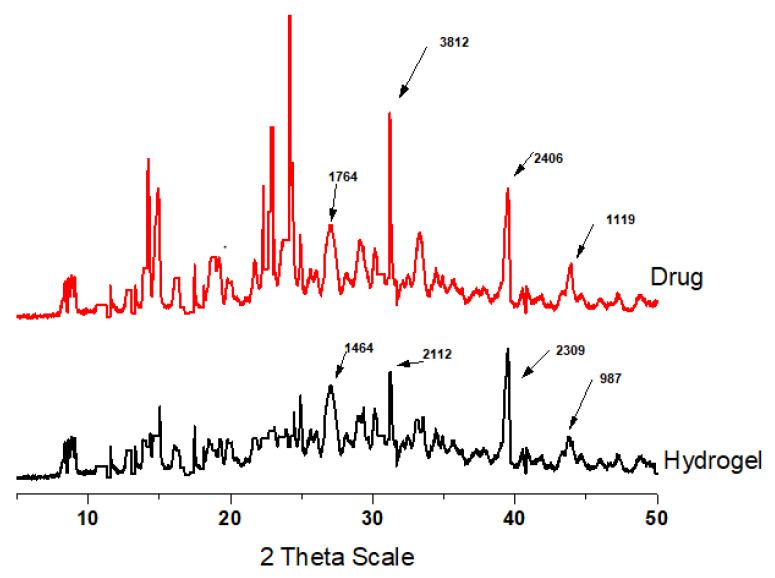
PXRD of drug (MPT) and IPN hydrogel.

Like a person’s fingerprint, the crystallographic pattern of a substance can be used to uniquely identify it. For any given substance, the crystallographic pattern will always be the same, but in a mixture, each component will generate its own unique pattern. To confirm the amorphous or crystalline nature of the MPT samples, PXRD of the MPT and IPN hydrogel formulations was carried out. The drug’s crystalline nature is seen in its strong peaks at 2θ = 07.5°, 15.11°, 24.15°, 26.24°, 32.16°, 39.35°, and 43.32° (Figure 5). The presence of typical MPT peaks (2θ = 26.12°, 32.16°, 39.24°, and 43.41°) in the loaded IPN hydrogel X-ray diffraction pattern suggests that the MPT crystalline state has slightly decreased, which may be due to the dilution factor of MPT within polymers or a decrease in the crystallinity of the drug in the drug delivery system. The appearance of characteristic peaks in the hydrogel indicated that there were no chemical interactions or pharmaceutical incompatibilities between the drugs and polymer excipients in a single-dosage form. Such symptoms foretell the distribution of medications in the IPN that satisfies the requirements for the improved drug delivery system (DDS).

### 2.7. Determination of Gel%, Yield%, and Gel Time

**Figure 6:**
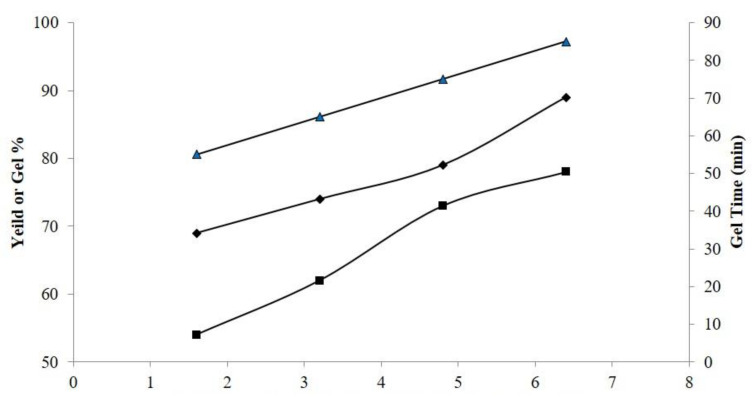
Effect of HPMC-co-poly(MAA)/Neocel C19 IPN hydrogels on gel%, yield%, and gel time.

The effect of different components on the gel percentage, yield percentage, and gel time of HPMC-co-poly(MAA)/Neocel C19 IPN hydrogels is shown in Figure 6. The yield and gel percentages increased with increasing Neocel C19 and HPMC concentrations. Increased polymer concentration (Neocel C19 and HPMC) may have made more radicals available for polymerization [27]. The formulation use of a specified amount of MBA to crosslink Neocel C19 and HPMC with MAA (monomer) may be the cause of the longer gelling time. A greater reaction (polymerization) rate could be the cause of the shorter gelling time [28]. These results are similar to those of previously reported studies.

### 2.8. Swelling Study

**Figure 7:**
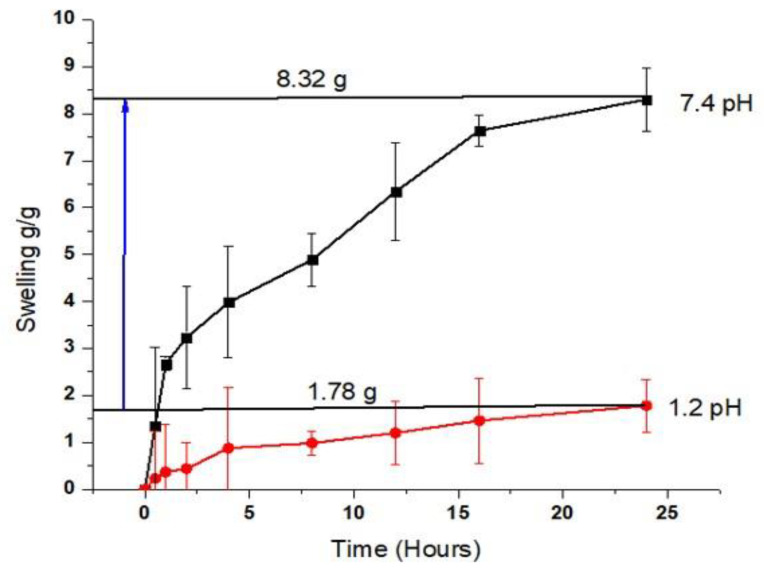
Swelling index of the IPN hydrogel (NH-5) at pH 1.2 and pH 7.4.

Hydrogels were proved to be pH sensitive as a remarkable dynamic swelling response ranging within 8.32 g/g was noticed at pH 7.4 as compared with pH 1.2. This test was run to evaluate the effects of various polymeric network components on the ability of the IPN hydrogel to swell. IPN swelling was higher at pH 7.4 compared with pH 1.2, where there is low ionization, since there is more functional group (carboxyl) ionization there (Figure 7). A formulation with a higher amount of Necocel C19 has a higher swelling property because more inter- and intramolecular self-crosslinkages with other polymers occur in the presence of a monomer in pH 7.4. Ion repulsion, triggered by complete ionization, leads to an expansion in size known as swelling [29]. Hydrogels swell and open pores when their COOH groups, which are added by MAA, are deprotonated at a basic pH. At an acidic pH, these functional groups become protonated, leading to decreased repulsive forces of polymeric chains and hydrogel deswelling.

**Figure 8:**
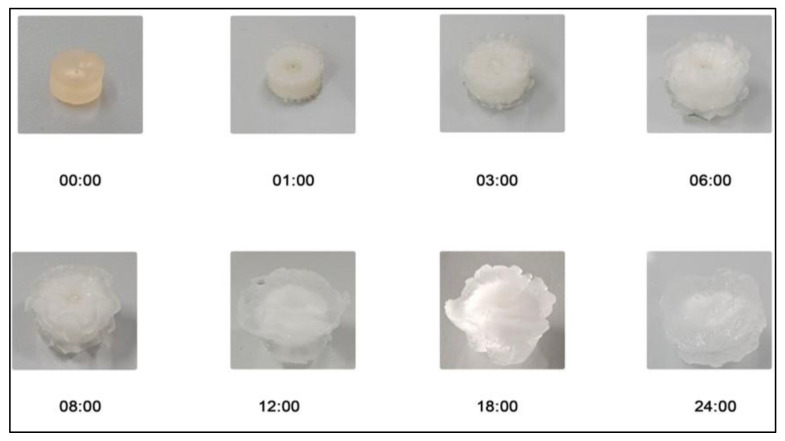
Effect of pH 7.4 along with time on swelling of IPN hydrogels.

The graph shows that increasing the concentration of Neocel C19 caused the swelling of IPN hydrogels to increase (Figure 8). This increase in swelling is presumably because more radicals are available due to an increase in polymer concentration [30]. Neocel C19 also alters the degree of crosslinking and carboxymethylation, which results in hydrophilicity by weakening hydrogen bonds and allowing water to invade molecules [31]. Increased HPMC content causes edema in the dead. HPMC quantity and viscosity directly correlate with swelling. A more viscous solution results from increasing the HPMC concentration, which raises the density. Compact polymer structures prevent the solvent from being absorbed, which reduces swelling [32].

### 2.9. Misoprostol (MPT) Release Study

**Figure 9:**
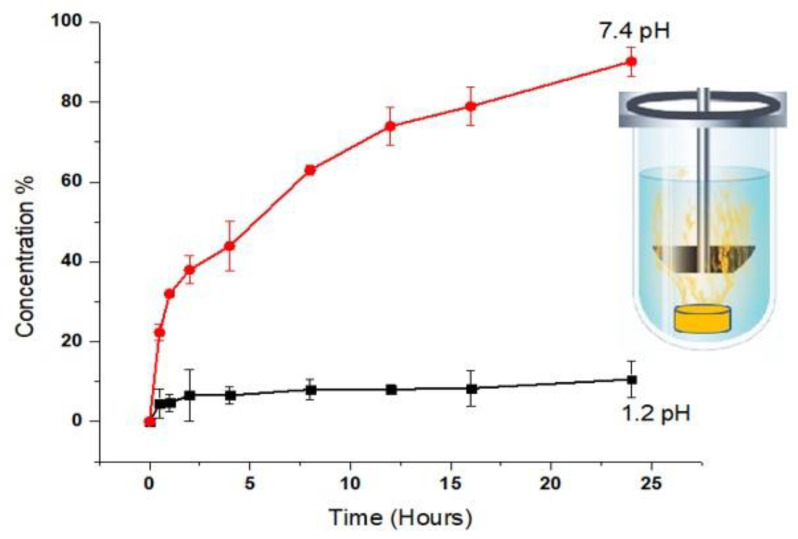
The percentages of drug release from the hydrogel (NH-5) at pH 1.2 and pH 7.4.

By conducting dissolution at pH 1.2 and 7.4 for 24 h at various intervals, the release profile of the MPT from IPN hydrogels was obtained (Table 3). Sample absorbance measurements were made using HPLC (Shimadzu, Germany) at a specific wavelength (maximum 200 nm). IPN hydrogels exhibit higher MPT release at pH 7.4 (80% in 20 h), which may be caused by the ionization of the R-COOH (carboxylic) group of MAA. Ionization produces repulsion, which opens spaces for the uptake of water, disc expansion, and subsequent MPT release [33]. In the first five hours, 40% of the drug was released, which is mandatory for the loading dose. The rest of the drug was released in a controlled manner. The same results were shown in a swelling study in which 8.32 g of hydrogels swelled within 20 h. Nevertheless, under pH 1.2 conditions, the release of MPT in IPN hydrogels was found to be diminished due to the protonation of COOH groups. This protonation decreased the repulsion within the IPN hydrogel, resulting in reduced swelling of the discs [34]. Figure 9 illustrates the percentages of MPT release for NH-5 at pH 1.2 and pH 7.4.

### 2.10. Hemolysis Assay

**Figure 10:**
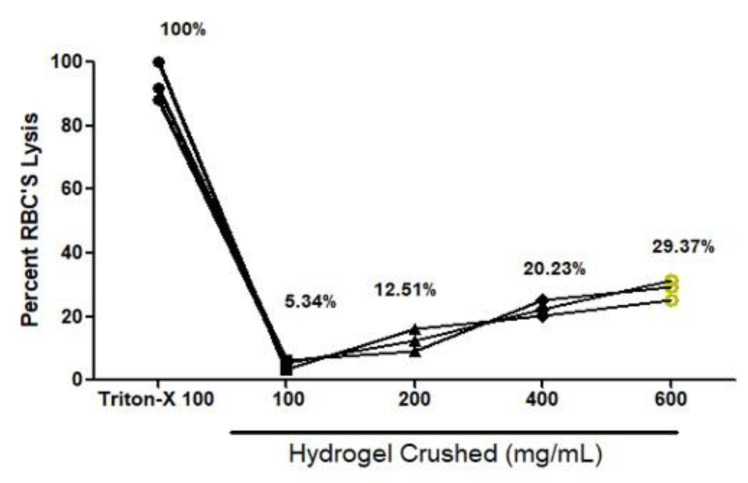
Hemolysis graph of IPN hydrogel (NH-5).

IPN hydrogel compatibility with blood was tested using a quick and accurate approach called the hemolysis assay. The hemolysis assay measures erythrolysis and hemoglobin dissociation when blood comes into contact with hydrogels. Different weights in mg of crushed hydrogel were taken for the hemolysis study (Figure 10). After that, a spectrophotometer was used to measure blood compatibility. A graph was created using PBS as the negative control and Triton-X as the positive control (which had 100% lysis). The blood compatibility of the IPN hydrogel was measured using different weights (100 mg, 200 mg, 400 mg, and 600 mg; 5.34%, 12.51%, 20.23%, and 29.37%, respectively). Each lysis percentage was found to be less than 50%, and for 100 and 200 mg, it was found to be very low at 15% (Figure 10). According to our hemolysis analysis, hydrogels could not significantly trigger hemolysis. This might be a result of the hydrophilic characteristics and blood compatibility of both Necocel and HPMC polymers. Because of its flexible mechanical, physical, and biological features, our results suggest that the HPMC/Necocel IPN hydrogel has long-lasting hemostatic potential for usage. The results also show that this hybrid system is more suitable for delivering MPT without any issues.

### 2.11. In Vivo Pharmacokinetic Study

**Figure 11:**
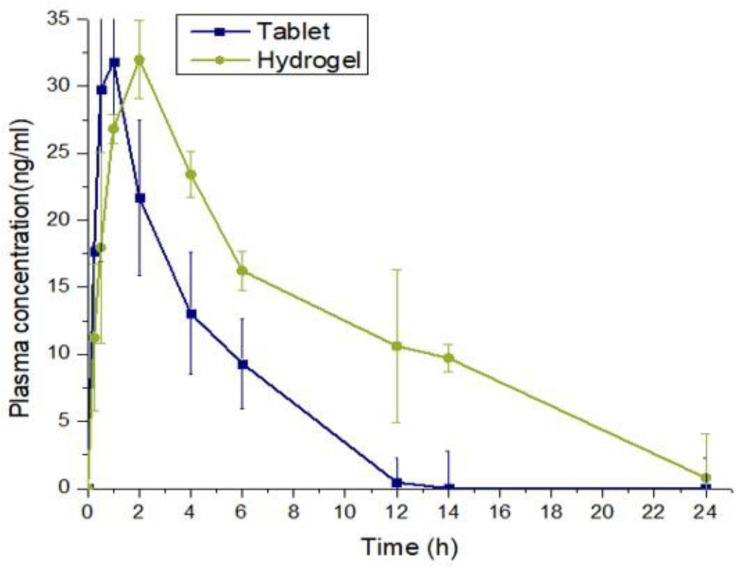
Plasma concentration graph of tablet and IPN hydrogel.

To learn more about the sustained release response from the grafted network, pharmacokinetic tests were performed on healthy rabbits. For this aim, oral solutions of the optimal hydrogel formulation (NH-5) and tablet were administered to both groups of animals (B and C) at equivalent doses, followed by 5–10 mL of water. Values for the pharmacokinetic parameters are shown in Table 4, and Figure 11 compares the plasma profiles of the tablet and IPN formulations. Plasma drug concentrations were found at predetermined time intervals. Due to the drug’s quick absorption, the maximum concentration of the tablet (Cmax) in the oral solution was found to be 30.44 ng/mL. For the formulation of hydrogels, these parameters were discovered to be 31.84 ng/mL. The drug’s half-life was 8.26 h, but when it was given as hydrogels, it was extended to 10.70 h. The gradual release of misoprostol from the polymeric matrix, which results in its sustained action in plasma and enhanced bioavailability, can be used to explain the prolonged half-life. Additionally, a substantial difference was found between the pure drug and the crosslinked network in terms of other pharmacokinetic characteristics. The tablets’ MRT was 13.17 h, which was decreased in hydrogels, and its AUC was increased from 314.41 ng h/mL to 400.50 ng h/mL in hydrogels. Hence, as demonstrated by the in vivo investigations, the IPN network system proved to be a good and effective platform for delivering and extending the release of misoprostol. The pharmacokinetic properties of the drugs showed improvement in our work with hydrogel. Additionally, we did not observe any adverse effect in the hydrogel group throughout the experiment. The rabbits remained healthy after the completion of the study. No weight loss was observed in any group and no suspicious movement was recorded during the study. 

### 2.12. Stability Study

**Figure 12:**
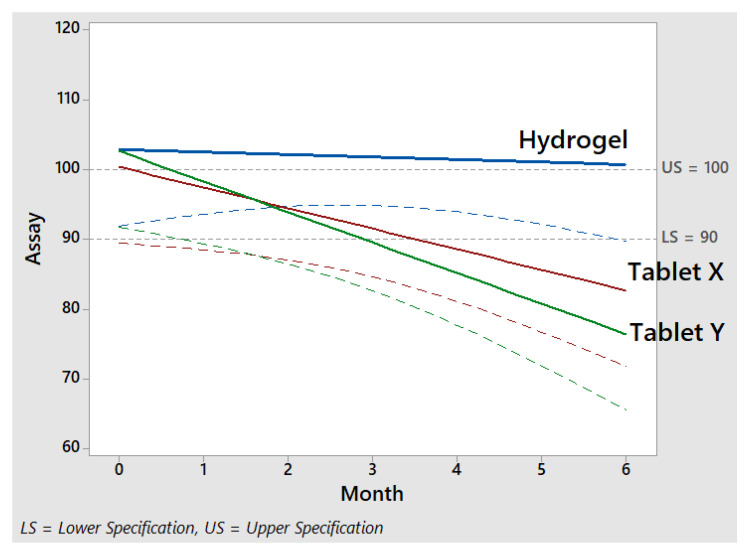
The stability comparison of market tablets and IPN hydrogel in terms of assay.

The outcomes of the accelerated stability study of the successful formulation NH-5 in terms of the assay are shown in Figure 12. The comparison of market tablets and IPN hydrogel was determined on an assay basis. We purchased two brands from the market (X and Y) for which the manufacturing dates were very recent. We performed the initial assay of marketed tablets (100.28 and 99.30%) and IPN hydrogels (101.93%) and kept the samples in a stability chamber and subjected to accelerated conditions, including a temperature of 40 °C and humidity of 75%, for a duration of 3 months. The assay profiles of the marketed tablets and IPN hydrogels were comparable after storage for 3 months, which was the shelf life calculated in accordance with US Food and Drug Administration criteria. The results for the IPN hydrogels were very appreciable in comparison with those for the marketed tablets. In the stability trials, the drug content of the tablets was 82.32 and 73.35, and for the IPN hydrogels, it was 100.71. This indicates that no substantial degradation occurred in the IPN hydrogels during that time.

## 3. Conclusions

IPN hydrogels based on HPMC/Neocel C19 were successfully created using FRPM. Neocel C19 and HPMC were crosslinked with MAA via MBA. pH sensitivity was demonstrated by traits such as swelling and drug release from IPN hydrogels. FTIR, SEM-AFM, DSC, and TGA were used to evaluate the IPN hydrogel’s characteristics. Studies on drug liberation in vitro revealed a direct correlation between drug release and IPN hydrogel swelling. The pharmacokinetic results also indicated that the IPN hydrogel has a more extended release throughout a period of 24 h. The tablet MRT was 13.17 h, which was decreased in hydrogels, and its AUC was increased from 314.41 ng h/mL to 400.50 ng h/mL in hydrogels. The blood compatibility of the IPN hydrogel was measured using different weights (100 mg, 200 mg, 400 mg, and 600 mg; 5.34%, 12.51%, 20.23%, and 29.37%, respectively). IPN hydrogels exhibit higher MPT release rates at pH 7.4 (80% in 20 h). In the stability trials, the drug content of the tablets was 82.32 and 73.35, and for the IPN hydrogel, it was 100.71. This indicates that no substantial degradation occurred in the IPN hydrogels during that time. All of the results point to HPMC-co-poly(MAA)/Neocel C19 IPN hydrogels being suitable carriers for controlled misoprostol release during treatment, and they also show that they are stable in hydrogel form. Patients will benefit from this predetermined drug combination with a polymeric network, and due to its prolonged drug release, less frequent dosing will be needed.

## 4. Materials and Methods

### 4.1. Chemicals

Saffron Pharmaceutical Faisalabad, Pakistan, provided misoprostol (MPT) for research purposes with 99% purity. HPMC K100, Neocel C91, and methacrylic acid (MAA) were purchased from Sigma-Aldrich (St. Louis, MO, USA). Sigma-Aldrich (St. Louis, MO, USA) also provided ammonium peroxodisulfate (APS), methylenebisacrylamide (MBA), and sodium hydrogen phosphate (SHP). Marketed tablets were purchased from a local brand (Nova Care) in Pakistan. 

### 4.2. Synthesis

**Figure 13:**
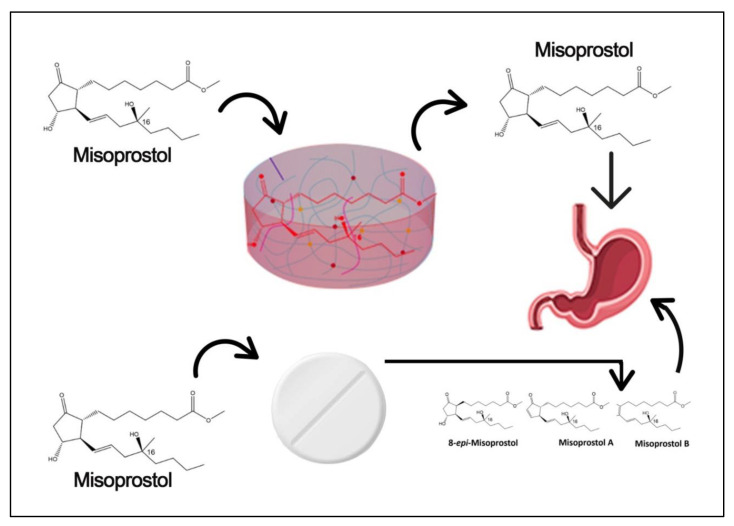
Differences in hydrogels and marketed tablets regarding drug stability.

Hydrogels were prepared by using the unique combination of HPMC/Neocel C19 with a free radical polymerization process. In Table 5, the concentrations of both polymers are mentioned. Neocel C19 and HPMC K100 polymers were separately weighed, added to a beaker, and mixed with deionized water. Polymers were dissolved by stirring (1500 rpm) at room temperature (25 °C) ± 2. Both polymer solutions were mixed at the same temperature to form a single polymer solution. Drop by drop, the monomer (MAA) was added to the aforementioned polymer solution after being precisely weighed according to Table 5. To start the polymerization reaction, APS was added to water and mixed with HPMC/Neocel C19 and MAA solution. Finally, the crosslinker (MBA) was dissolved in water to make a solution, and this solution was slowly added to the reaction solution (Figure 13). The finished solution was additionally ultrasonically blended to achieve full mixing. To release any trapped oxygen, a stream of nitrogen was given through the reaction mixture. The final solution was poured into the test tubes, which were then wrapped in aluminum foil and heated in a water bath at 50 °C ± 2 for 24 h. The test tubes were removed after the stipulated amount of time had passed, and the cylindrical gel was gathered. The gel was then divided into pieces that ranged in size from 6 to 8 mm in diameter and thickness of the hydrogels was maintained at 2 mm. These hydrogel discs were kept in Petri dishes. To completely remove all unreacted monomers, the cylindrical discs were then immersed in an aqueous solution of ethanol (50% *v*/*v*). Throughout the rigorous washing of the gels, the pH of the washing solution was closely observed. Discs were dried at room temperature after being baked in an oven (50 °C ± 2) until a homogenous mass was obtained. The discs were then removed from the oven and stored [35]. Five different formulations were prepared by varying the Necocel C19 polymer. The remaining ingredients and polymer were the same throughout the experiment. We chose the optimal formulation and pursued characterizations on the basis of drug loading properties.

### 4.3. Loading of Misoprostol (MPT)

To carry out MPT loading, the IPN polymeric network was submerged in a solution of MPT in water for five days. After allowing the hydrogel to reach equilibrium, the disc was removed, dried at room temperature, and then placed in the oven to obtain a consistent mass [36].

### 4.4. Determination of Drug Entrapment Efficiency

MPT-loaded hydrogels were broken/crushed to evaluate drug entrapment. Hydrogel that had been crushed and weighed was soaked in phosphate buffer (25 mL) at pH 7.4 for 24 h. We used the method as mentioned in United States Pharmacopeia (USP Pharmacopeia). To extract MPT from crushed hydrogel, a 20 min sonication procedure was performed. To measure the amount of MPT, the clear supernatant solution was analyzed with the mobile phase using HPLC with the maximum wavelength set at 200 nm [21]. It was estimated that the entrapment efficiency of prepared hydrogels for MPT might be represented by the following equation [22].

(1) $$\mathrm{Entrapment}\;\mathrm{efficiency}\%=\frac{\mathrm{Actual}\;\mathrm{drug}\;\mathrm{content}\;\mathrm{in}\;\mathrm{IPN}\;\mathrm{hydrogel}\;}{\mathrm{Theoretical}\;\mathrm{drug}\;\mathrm{content}\;\mathrm{in}\;\mathrm{IPN}\;\mathrm{hydrogel}}\times 100$$

### 4.5. Method Development and Optimization for Pharmacokinetic Study

MPTs are UV-active chemicals because they contain benzene rings and conjugated groups in their structures. Maximum absorbance was measured at a wavelength of 200 nm. The reference standard of MPT was run with a concentration of 10 µg/mL to detect the wavelength. A Shimadzu LC 20AB HPLC system (Tokyo, Japan) with a 1000 pump and UV-VIS detector was used in this analysis. The mobile phase consisted of a mixture of acetonitrile and sodium acetate buffer with a pH of 3.2. The stationary phase consisted of an Agela C18 column that was 250 millimeters by 4.6 millimeters and had a particle size of 5 micrometers. The injection volume for HPLC analysis was kept at 20 µL, along with the 1.0 mL/min flow rate, and the UV detector was set to a wavelength of 200 nm. The acquired chromatograms were compared with the reference standard of MPT, and the drug loading efficiency was calculated in triplicate. The proposed technique was effectively used to investigate drugs in plasma samples [37]. We carried out this assay in rabbit plasma under identical conditions, and the outcomes demonstrated its validity as a technique. According to USP and ICH guidelines, the analytical process was carried out, and numerous parameters, including precision, accuracy, specificity, and linearity, as well as the limit of quantification (LOQ) and limit of detection (LOD), were determined. 

### 4.6. Plasma Sample Pretreatment

Blood samples from rabbits were collected and stored at −70 °C. Next, 20 µL of a drug solution with a known concentration that had been made through a series of dilutions was added to 100 µL of plasma solution that had been collected into a plastic tube. To precipitate the protein, we next added 50 µL trichloroacetic acid (TCA) to the drug and plasma solution and started centrifugation. The supernatant was collected after centrifugation (15,000× *g*, 15 min, 30 °C) and added to the mobile phase (100 µL) for HPLC analysis.

### 4.7. Scanning Electron Microscopy (SEM)

SEM was used to examine the surface morphology of IPN polymeric networks. A sample of hydrogel cut into pieces of the desired size was placed on an aluminum mount and sputtered through gold and palladium. For scanning samples, a 20 kV accelerated voltage with a 5–15 mm space gap was used [38].

### 4.8. Fourier Transform Infrared Spectroscopy (FTIR)

IPN hydrogels were crushed to the required size to determine drug compatibility and interaction. FTIR analysis of the polymers, drug, and hydrogel discs was performed. With the use of software called OPUS data collection and Bruker FTIR (Tensor 27 Series-Bruker Corporation, Germany) equipment, a value range of 4000 to 800 cm^−1^ for spectrum scans was achieved [39,40,41].

### 4.9. Thermal Analysis

With thermal analysis equipment, TGA and DSC of the polymers, formulation, and drug were performed (TA instrument Q2000 Series, West Sussex, UK). For TGA and DSC, adequate sample heating was carried out in a N2 environment at 10 °C per minute up to 400 °C [42,43].

### 4.10. Powder X-ray Diffraction

Utilizing PXRD, the composition of IPN hydrogels was studied. A powder X-ray diffractometer was used to examine the samples (x-Pert—PAN analytical, The Netherlands). The angle of diffraction ranged from 10 to 40° [44,45,46,47].

### 4.11. Determination of Gel%, Yield%, and Gel Time

The amount of reactant polymerization during the preparation of hydrogels was calculated using the formulas gel% and yield%. The dried hydrogel was macerated in water for seven days and stirred occasionally to eliminate polar components. Equations (2) and (3) were used to calculate the yield percent after drying the water-immiscible component of the polymeric network in an oven to form a permanent weight (m_d_) Gel.

(2) $$Gel\%=\frac{m_{d}}{m_{i}}\times 100$$

(3) $$Yield\%=\frac{m_{d}}{m_{c}}\times 100$$

### 4.12. Swelling Study

The manufactured hydrogel was weighed before being placed at a temperature of 37 degrees Celsius in 0.1 M HCl solution with a pH of 1.2 and 0.2 M phosphate buffer solution with a pH of 7.4 in order to analyze the dynamic swelling and the impact of pH sensitivity. Hydrogel was frequently removed from the media that caused swelling to weigh the swelled discs at predetermined intervals until a constant and uniform weight was achieved. Using the formula below, we were able to compute the normalized swelling degree Q at time t in terms of grams of water per gram of dry gel [48].

(4) $$\;Q_{t}=\frac{\begin{array}{cc} m_{t} & -m_{o} \end{array}}{m_{o}}$$

where M_0_ = weight of IPN hydrogel before swelling, m_t_ = weight of IPN hydrogel after swelling (dry gel), and Q_t_ = weight of water absorbed.

### 4.13. Release Study

In order to measure drug release, the USP Dissolution Apparatus II was utilized at both an acidic pH of 1.2 and a basic pH of 7.4. A steady level of drug concentration was retained inside the dissolving media by continuously spinning the polymer disc at a rate of fifty revolutions per minute after it had been weighed and added to 900 milliliters of the medium. The temperature of the dissolving medium was set to 37 °C. Up to 24 h later, samples were collected at predetermined intervals. Fresh medium was added each time the sampled volume was changed. The release of MPT was measured at a wavelength of 200 nm [49]. The following formula was used.

(5) $$\%\;\mathrm{Release}=\frac{\mathrm{Absorbance}\;\mathrm{of}\;\mathrm{the}\;\mathrm{sample}\;\mathrm{solution}\;}{\mathrm{Absorbance}\;\mathrm{of}\;\mathrm{the}\;\mathrm{standard}\;\mathrm{solution}}\;\times 100$$

### 4.14. Determination of Pharmacokinetic Parameters

According to the guidelines of a specific process, the pharmacokinetic parameters were estimated using the MS-Excel 2010 program from the plasma drug concentration data at various time points. Pharmacokinetic tests for marketed tablets and hydrogels were carried out in rabbits, which were divided into three groups (A, B, and C) (A: marketed tablets, B: hydrogels, and C: control group). These animals (n = 6) weighed more than 1 kg and were housed in cages under regulated conditions with free access to food and water. They were made to fast for the duration of the trial. Crushed tablets (2.8 µg of API) and hydrogels (2.8 µg equivalent to misoprostol) were given orally to rabbits. Blood samples (100 µL) were subsequently taken at predefined intervals in heparinized tubes. Plasma was collected in Eppendorf tubes and stored at −70 °C after centrifugation at 4000 rpm for 15 min. The substance was extracted using a liquid-liquid extraction process. After being vortexed for 5 min and then centrifuged at 4000 revolutions per minute for 15 min, the mixture was subjected to these processes in order to separate the organic solvent from the supernatant. The oven was used to dry the divided layer. Next, a Promosil C18 column was used to inject the dried residue into the RP-HPLC system at a flow rate of 1 mL/min after being diluted with mobile phase and vortexed. A program named PK Solver was used to determine the pharmacokinetic parameters. The pharmacokinetic study was authorized by the RLCP, Lahore, Pakistan’s Ethics Committee (study no.: RLCP-19641-2022).

### 4.15. Hemolytic Investigations

The supernatant was discarded, and the precipitate was washed in phosphate-buffered saline (PBS) three times after being placed in a tube containing ethylene diamine tetra acetic acid and spun at 1500 rpm for 5 min to perform the hemolytic test on human blood. A total of 200 mL of washed blood sediment was mixed with 4 mL of phosphate-buffered saline and then vortexed for a while. The mixture was centrifuged at 1500 rpm for 5 min after the samples were kept at 37 °C for 5 h, and 541 nm wavelengths were used. In this experiment, the negative control was phosphate-buffered saline, and the positive control was Triton X-100. Both of these were utilized as the standard variables. The abnormality of blood cells was observed through UV absorbance, and % hemolysis was determined by using Equation [50].

(6) $$\%\;\mathrm{Hemolysis}=\frac{\mathrm{Absorbance}\;\mathrm{of}\;\mathrm{sample}-\mathrm{Absorbance}\;\mathrm{of}-\mathrm{ve}\;\mathrm{control}}{\mathrm{Absorbance}\;\mathrm{of}+\mathrm{ve}\;\mathrm{control}-\mathrm{Absorbance}\;\mathrm{of}-\mathrm{ve}\;\mathrm{control}}\times 100$$

### 4.16. Stability Study

The tablets were purchased from a market (X and Y) and were stored for up to three months at 40 2 °C/75 5% relative humidity after initial testing. IPN hydrogels were also treated as we mentioned for tablets. IPN hydrogels were kept in glass bottles with sealed caps. After three months, all products were withdrawn from the chambers, and the assay was performed. A graph was made by using Minitab software.

### 4.17. Analytical Statistics

The statistical analysis was carried out with the help of the Graph-Pad Prism v.5 program. The analysis consisted of one-way ANOVA as well as Tukey’s test. The mean and standard deviation were used to provide a representation of the data (SD). As a threshold for statistical significance, the *p* statistic value of 0.05 was selected.

## 5. Future Research

The controlled release of many therapeutic compounds that are water-soluble has been made possible by conventional hydrogels, but entrapment of hydrophobic medicinal molecules has proven difficult. There are maximum numbers of poorly water-soluble molecules that are encapsulated with other techniques to target and regulate the drugs. The hydrogel approach can be used for these types of agents because the hybrid system is more efficient and easier to use. 

## Figures and Tables

**Table 1:** Drug entrapment efficiency (%) of formulations.

Formulation	Drug Entrapment Efficiency (%)
NH-1	64.21
NH-2	69.16
NH-3	73.21
NH-4	75.26
NH-5	82.31

**Table 2:** Regression analysis and sensitivity determination along with LOD and LOQ.

Parameters	MPT
Working λmax	200
Regression equation	0.0019 × 0.0032
R_2_	0.996
LOD μg/mL	5 ng/mL
LOQ μg/mL	10 ng/mL
Retention time (min)	6.97
Recovery	92.2% ± 3.1%, 89.2% ± 2.0%, and 89.5% ± 3.2%
Accuracy	94.1% to 102.3% and 96.2% to 97.4%
Tailing factor	1.12 ± 0.2
Asymmetry	0.78 ± 0.07

**Table 3:** The drug entrapment efficiency for all formulations and the percentages of drug release at pH 1.2 and pH 7.4 for optimized formulation (NH-5).

Formulation	Drug Entrapment Efficiency %	Percent Released (24 h Period)	
		pH 1.2	pH 7.4
NH-1	64.21		
NH-2	69.16		
NH-3	73.21		
NH-4	75.26		
NH-5	82.31	10.8	90.23

**Table 4:** Pharmacokinetic parameters of tablet and hydrogel after single-dose oral administration to rabbits.

Parameter	Tablet	Hydrogel
*t_1/2_* (h)	8.26 ± 0.185	10.70 ± 0.53
*AUC _0-t_* (ng/mL*h)	314.41 ± 1.93	400.50 ± 4.19
*T_max_* (h)	2	1.5
*C_max_* (ng/mL)	30.44 ± 0.38	31.84 ± 0.014
*AUC _0-inf_* (ng/mL*h)	340.61 ± 2.39	488.20 ± 4.53
*λz* (l/h)	0.094 ± 0.011	0.089 ± 0.0021
*MRT 0-inf_obs* (h)	13.17 ± 0.12	11.48 ± 0.011

**Table 5:** Compositions of formulations, highlighting the key ingredients and their proportions.

Formulation No.	% Wt				
	Neocel C19	HPMC K100	MAA	APS	MBA
NH-1	0.4	0.4	24	0.32	0.4
NH-2	0.8	0.4	24	0.32	0.4
NH-3	1.2	0.4	24	0.32	0.4
NH-4	1.6	0.4	24	0.32	0.4
NH-5	2.0	0.4	24	0.32	0.4

## Data Availability

All the raw data in this research can be obtained from the corresponding authors upon reasonable request.
